# Concomitant Granulomatosis with Polyangiitis and C3 Glomerulonephritis Causing Renal Failure

**DOI:** 10.7759/cureus.482

**Published:** 2016-02-05

**Authors:** Aadel A Chaudhuri, Jason T Davis

**Affiliations:** 1 Department of Radiation Oncology, Stanford University School of Medicine; 2 Department of Nephrology, Scripps Mercy Hospital

**Keywords:** c3 glomerulonephritis, c3gn, gpa, renal failure, vasculitis, autoimmunity, wegener's granulomatosis, granulomatosis with polyangiitis

## Abstract

A 75-year-old male with no prior history of vasculitis or renal deficiency presented with a history of 36 hours of anterior epistaxis, one month of worsening shortness of breath, severe anemia requiring transfusion, thrombocytopenia, coagulopathy with INR 4.9, and renal failure requiring hemodialysis. A peripheral blood smear revealed no evidence of microangiopathic hemolytic anemia. We performed an autoimmune workup, which revealed high levels of serum serine protease 3 antibody (C-ANCA), elevated rheumatoid factor, low serum C3, and normal levels of serum C4. We performed a renal biopsy and then performed light microscopy, immunofluorescence, and electron microscopy on the resulting samples. This revealed that approximately half of the sampled glomeruli were globally sclerotic, consistent with severe renal disease. Among the non-sclerotic glomeruli, several demonstrated diffuse granular mesangial staining for C3, while other glomeruli had small crescents, consistent with a mixed picture of C3 glomerulonephritis (C3GN) and crescentic glomerulonephritis. The patient responded well to treatment with cyclophosphamide and prednisone, with a resolution of his acute issues, significant improvement in kidney function, and was eventually weaned from routine hemodialysis. In summary, this is a unique case of a patient presenting with features of both granulomatosis with polyangiitis (GPA) and C3GN.

## Introduction

Granulomatosis with polyangiitis (GPA), formerly known as Wegener’s disease, is an anti-neutrophil cytoplasmic antibody (ANCA) associated autoimmune vasculitis that occurs mostly in older adults, and typically presents with ear/nose/throat, pulmonary, and renal manifestations. Like other ANCA-associated vasculitides, GPA is characterized by pauci-immune glomerular deposits. A few reports have shown that ANCA-associated glomerulonephritis can be associated with immune complex deposits [1-4].

C3 glomerulonephritis is a rare ANCA-negative glomerulonephritis, characterized by immunohistochemistry showing C3 glomerular deposits and electron microscopy showing glomerular C3 deposits. Pathogenesis involves excess activation of the alternative complement pathway. In most patients, C3GN is associated with depressed serum C3 levels but normal C4 levels [5].

Here, we present a unique case of a patient with newly diagnosed end-stage renal disease with clinical, laboratory, and histological features consistent with a mixed picture of GPA and C3GN. Informed patient consent was obtained for his treatment.

## Case presentation

A 75-year-old Hispanic male with no prior history of vasculitis or renal deficiency presented to the emergency department with 36 hours of anterior epistaxis and one month of progressively worsening shortness of breath. His epistaxis resolved after undergoing anterior nasal packing in the Emergency Department. A CT scan revealed fluid overload with bilateral pulmonary edema. Serum laboratory tests revealed severe anemia (hemoglobin 4.4 g/dL), thrombocytopenia (platelets 101,000/μL), coagulopathy (INR 4.9), severe acute kidney injury (creatinine 8.7 mg/dL, blood urea nitrogen 140 mg/dL), critical hyperkalemia (potassium 7.6 mmol/L), and anion gap metabolic acidosis. Erythrocyte sedimentation (72 mm/hr) and C-reactive protein (31.8 mg/L) were elevated. Coagulopathy labs showed elevated D-dimer (793 ng/mL) and elevated fibrin split products (5-20 mcg/mL) with borderline fibrinolysis suggesting a disseminated intravascular coagulation etiology of his coagulopathy; however, fibrinogen levels were normal (276 mg/dL), and no schistocytes were visualized on peripheral blood smear examination. The patient underwent emergency hemodialysis and transfusion of packed red blood cells and fresh frozen plasma. Notably, his previous serum laboratory tests, including blood urea nitrogen (BUN) and creatinine from two years prior, were within normal limits.

Autoimmune workup was performed to determine the etiology of this patient’s disease. Results came back positive for high levels of serum serine protease 3 antibody (C-ANCA) (76 AU/mL; reference range: 0-19), rheumatoid factor (132 IU/mL; reference range: 0-15), and low serum C3 (57 mg/dL; reference range: 90-180) but normal serum C4 (15.9 mg/dL; reference range: 10-40). Anti-nuclear antibody (ANA) was positive by serum enzyme immunoassay, but negative by Hep2-ANA immunofluorescence. All other serum rheumatologic tests, including myeloperoxidase antibody (P-ANCA), anti-double-stranded DNA antibody, anti-Smith antibody, SSA antibody, SSB antibody, anti-ribonucleoprotein antibody, anti-cardiolipin antibody, and anti-glomerular basement antibody, were negative.

**Figure 1:**
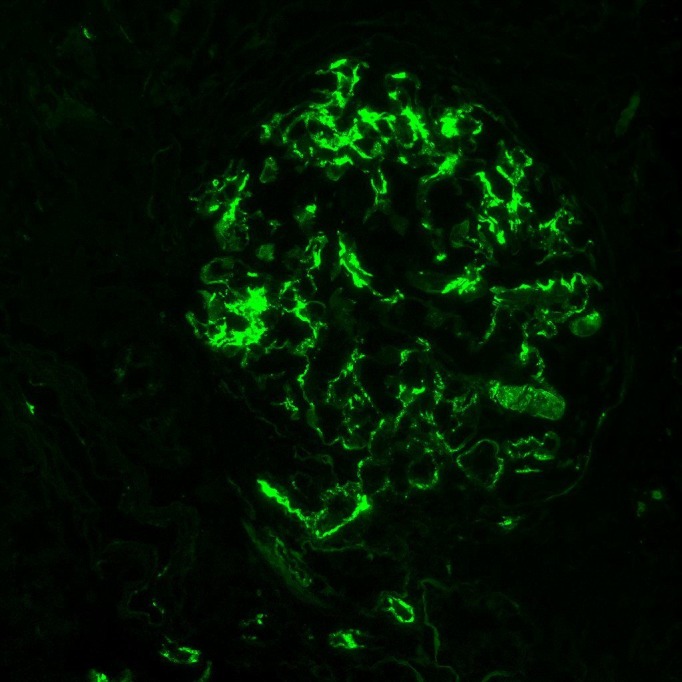
Glomerular immunofluorescence showing diffuse granular mesangial deposition of C3.

**Figure 2:**
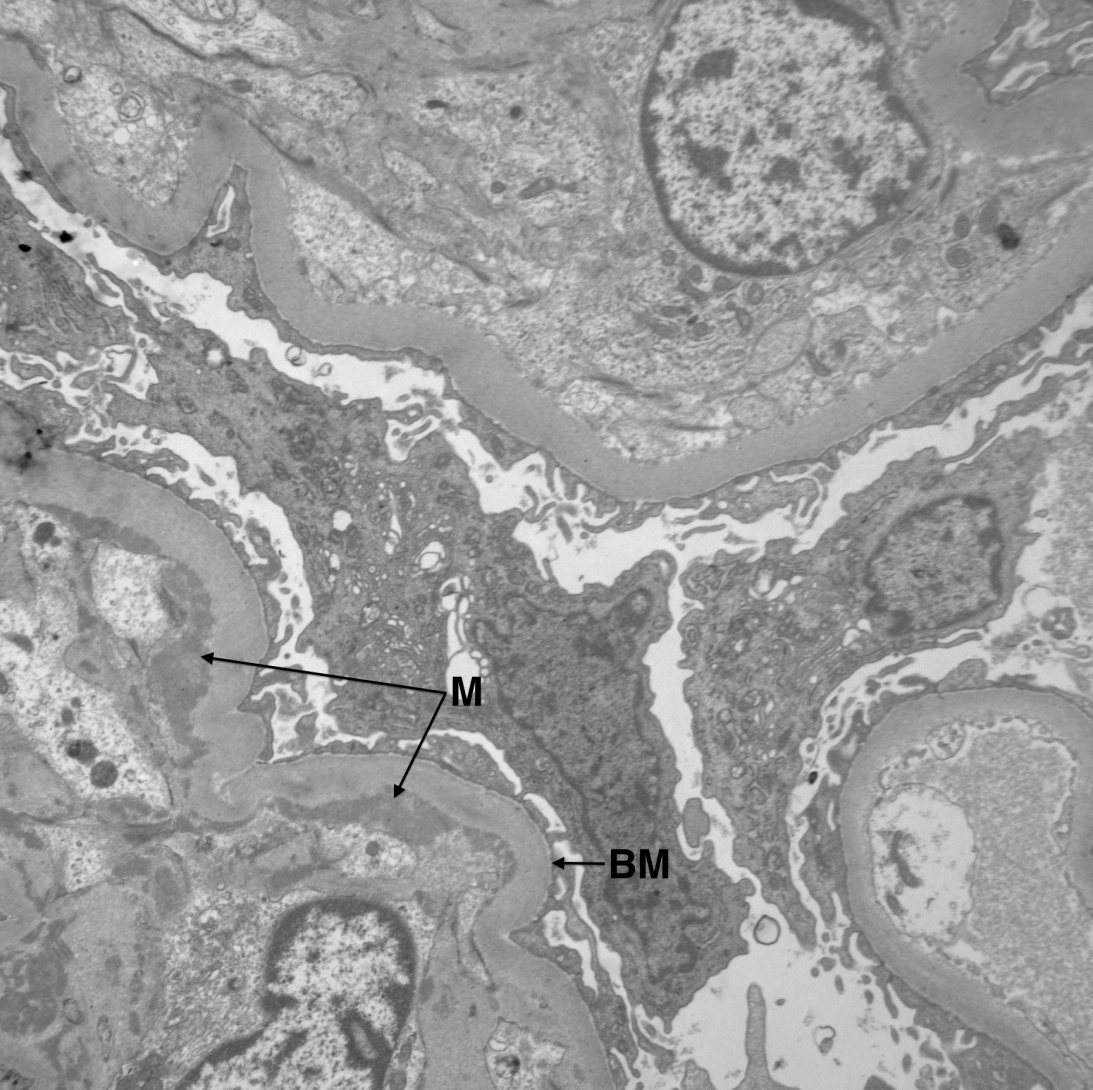
Electron microscopy demonstrating segmental subendothelial mesangial deposits (M) and glomerular basement membrane thickening (BM).

**Figure 3:**
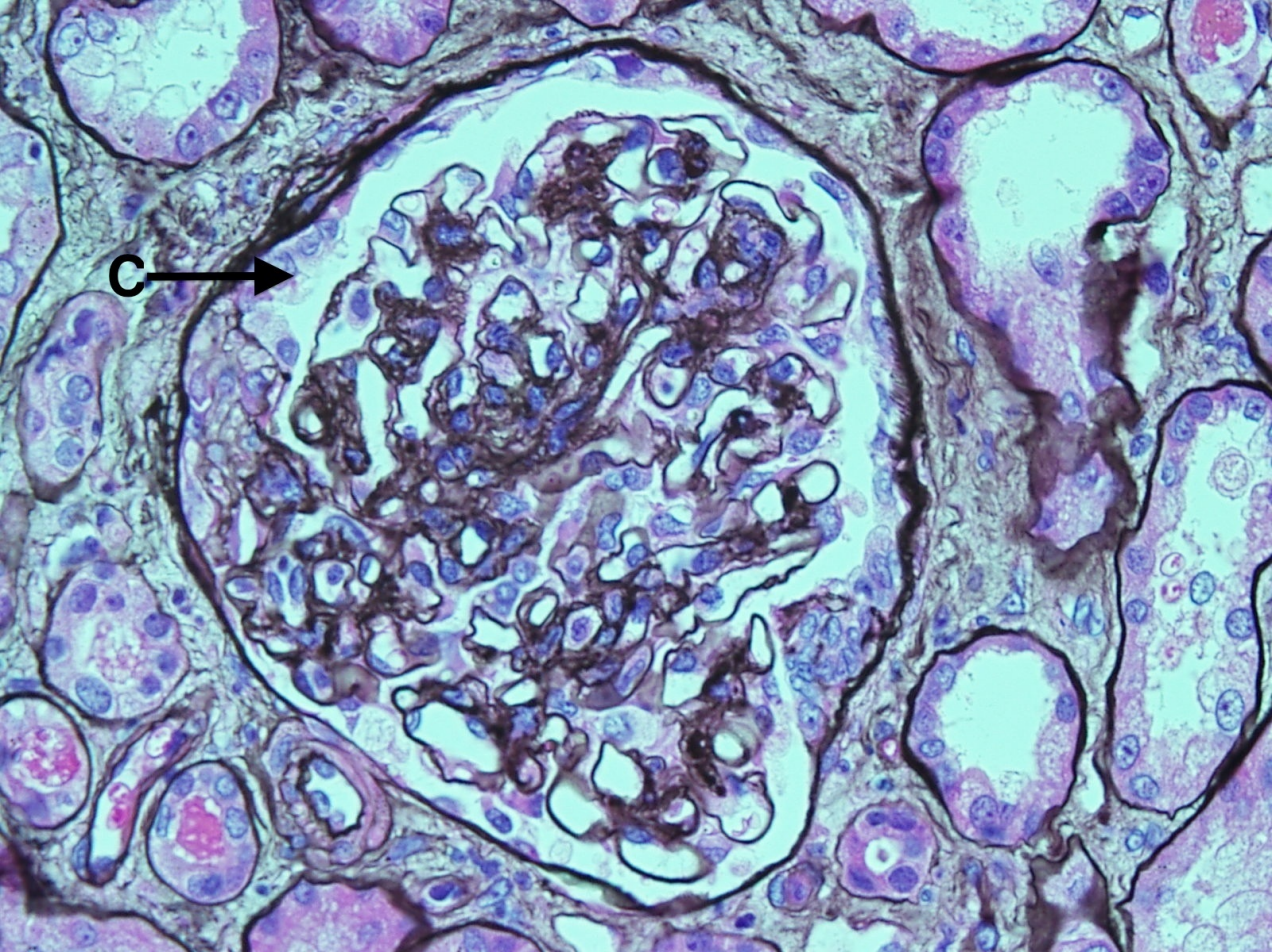
Light microscopy of a glomerulus showing segmental fibrocellular crescent (C).

The patient then underwent renal biopsy. Histopathology revealed that approximately half of the sampled glomeruli (9 of 17) were globally sclerotic, consistent with severe renal disease. Among the non-sclerotic glomeruli, the majority demonstrated diffuse, granular mesangial staining of C3 by immunofluorescence (Figure 1), with evidence of subendothelial mesangial deposits on electron microscopy (Figure 2). There were also some glomeruli with small crescents present (Figure 3). In short, pathology revealed a mixed picture of C3 glomerulonephritis and crescentic glomerulonephritis among the non-sclerotic glomeruli.

We treated the patient aggressively for what appeared to be a mixed picture of granulomatosis with polyangiitis (GPA) and C3 glomerulonephritis (C3GN) with prednisone, 60 mg PO daily, and cyclophosphamide, 150 mg PO daily, and administered hemodialysis three times per week. The patient tolerated the treatment well. After four months of treatment, the patient’s laboratory tests showed marked improvement in kidney function with a serum creatinine of 2.1 mg/dL, a BUN of 31 mg/dL, good urine output, and a 24-hour urine creatinine clearance of 24 mL/min. Thus, we were able to taper the patient’s doses of prednisone and cyclophosphamide, and discontinue hemodialysis. His serum creatinine level decreased further over the ensuing months and stabilized at 1.6 mg/dL.

## Discussion

GPA, like other ANCA-associated vasculitides, is typically associated with a pauci-immune glomerulonephritis. We note that recently some cases of ANCA-associated vasculitis have been reported where glomerular immune deposits were noted [1-4]. Notably, Hillhorst, et al. retrospectively analyzed 187 renal biopsies from ANCA-positive glomerulonephritis patients. Among these, 78 were found to stain positive for C3 complement by immunohistochemistry [2]. The majority of these patients did not have deposits noted on electron microscopy. Thus, without immunohistochemistry, they likely escaped detection of immune complexes prior to the authors’ analysis.

C3GN is a rare autoimmune disorder characterized by excess alternative complement pathway activation, with renal biopsy characteristic of glomerular C3 deposits, and most patients having depressed C3 levels but normal C4 levels [5]. Here, we presented a case with signs, symptoms, laboratory values, and histopathology consistent with both C3GN and GPA. It is unclear whether this patient truly harbored both diseases, which are typically caused by different immunologic pathways. It is possible that this patient harbored a variant of one of these diseases, likely GPA in light of its greater incidence in the population and other reports showing cases of GPA with glomerular immune deposits [1-4]. Still, this patient’s depressed serum C3 levels and normal C4 levels, diffuse glomerular C3 deposits on immunohistochemistry, and subendothelial deposits on electron microscopy strongly raise the possibility that this patient indeed had C3GN, in addition to his more definitively diagnosed GPA.

## Conclusions

To our knowledge, this is among the first reported cases of a patient presenting with signs, symptoms, laboratory values, and histopathology consistent with both C3GN and GPA. We showed that the patient responded well to treatment with prednisone and cyclophosphamide, with significant improvement in renal function and discontinuation of hemodialysis. GPA results from autoimmune lymphocytes within the adaptive immune system, leading to the production of pathologic C-ANCA autoimmune antibodies. C3GN typically arises from excess activation of the alternative pathway of the complement arm of the innate immune system. This patient’s unique presentation of both GPA and C3GN leading to renal failure, thus, may have been the result of aberrancies in both of the adaptive and innate arms of his immune system.
